# Reply to “Is the number of DNA repair genes associated with evolution rate and size of genomes?”

**DOI:** 10.1186/s40246-020-00261-9

**Published:** 2020-03-16

**Authors:** Konstantinos Voskarides

**Affiliations:** Medical School, University of Cyprus, Nicosia, Cyprus

**Keywords:** Adaptive radiation, Living fossils, Evolutionary stasis, Evolution rate, DNA repair genes, Genome

Dear Editor,

I appreciate the fact that our manuscript “Evidence that DNA repair genes, a family of tumor suppressor genes, are associated with evolution rate and size of genomes” [1] has gained attention by other scientists. Comments by Dr. I. Undroiu [2] are interesting but I think that some points of our work have been misunderstood. Below, I am answering to all the comments, one by one, since we believe that part of this critique is not solid.

1. The author claims that we had to follow the phylogenetic approach. We do not doubt that phylogenetic analysis is a reliable and a frequently used method in comparative genetics. On the other hand, this is not the only one approach. The aim of this study was not the phylogenetic relationships between species. Grouping of species was based on evolution rate following the terminology of “living fossils” or “radiated species.” We were looking for a common genetic pattern in one group, that not existing in the other group. We created a custom-made algorithm for this purpose (see Methods). The main question of this research was: “Is any gene family(ies) prominent in any of those two groups and why?”
2. The author claims that mammals have larger genomes and thus contain more genes than other taxa; this is why there is a positive linear regression between size of genomes and number of DNA repair genes. We believe that this aspect is false. It is widely known that size of genomes is not related with organisms’ complexity and number of genes, this being an old puzzle in genetics known as “C value” paradox [3, 4]. We also performed ourselves a linear regression to show this. On Fig. 2 of the original paper [1], it is obvious that the size of genomes is not linearly related with the number of encoded proteins.
3. The author claims that *MPG* gene should appear as an ortholog for *Elephantulus edwardii* since it shares high similarity with *MPG* of *Loxodonta africana*. Here, we would like to emphasize that this not the procedure that “NCBI Eukaryotic Genome Annotation Pipeline” follows. Orthologs are discovered by comparison with human genes, and gene names are given in compliance with specific criteria. Compare all by all is an endless and a wrong procedure. Additionally, regardless of similarity percentage, insertions, deletions, or frameshifts inside reading frames are taken into account for giving a formal gene name. Please visit the following NCBI web link for details: https://www.ncbi.nlm.nih.gov/genome/annotation_euk/process/
4. The author claims that some genes noted as absent in *Ornithorhynchus anatinus* genome (listed in supplementary data of our paper), are in fact present in NCBI list of orthologs. That is correct indeed. The reason is that NCBI databases are continuously updated and new data are introduced. In our published paper [1], we clearly state in Methods that “Genome and gene data used for this work are updated since April of 2019, according to Genome and Gene databases of NCBI.” After April of 2019, some new gene data have been added in NCBI. For the purpose of this letter, we investigated the new gene updates (after April of 2019) and we found that new gene predictions were added for five species out of the 44 analyzed in our paper. This can be easily confirmed in NCBI Gene Statistics webpage (https://www.ncbi.nlm.nih.gov/gene/statistics/?TAXORG=7742). We reanalyzed those five species’ updated gene files, and we found that *Delphinapterus leucas* has 1 more DNA repair gene, *Scleropages formosus* has 3 more DNA repair genes, *Geospiza fortis* has 4 more DNA repair genes, *Ornithorhynchus anatinus* has 23 more DNA repair genes, and that nothing changed for *Physeter catodon*. We then repeated *t* test statistics, and the result is still significant. Radiated species have more DNA repair genes in total (*p* = 1.1 × 10^−2^), more base excision repair genes (*p* = 7.7 × 10^−3^), and more nucleotide excision repair genes (*p* = 2.1 × 10^−3^) than living fossil species.

In our published paper [1], we cite other research works, e.g., in cetacean, where results are in accordance with our findings. Of course, we agree that our work is still preliminary, and more research is needed to be done by including more species or other methodologies. Genomes databases is a useful tool, but we have to anticipate the fact that genetic assembly is not perfect yet and that algorithms will continue getting improved the next few years.

## Data Availability

All data generated or analyzed during this study are included in this published article.
